# Primary Extra-nodal Diffuse Large B-cell Lymphoma of the Gingiva Mimicking a Dental Abscess: A Diagnostic Challenge

**DOI:** 10.7759/cureus.73349

**Published:** 2024-11-09

**Authors:** Anaïs Gommier, Loredana Radoi

**Affiliations:** 1 Oral Surgery, Faculty of Dentistry, Paris City University, Paris, FRA; 2 Oral Surgery, Louis Mourier Hospital, Colombes, FRA

**Keywords:** diffuse large b-cell lymphoma, gingiva, maxilla, neoplasm, oral cavity, primary non-hodgkin’s lymphoma, soft tissue

## Abstract

Lymphomas are malignant proliferations of B or T lymphocytes, classified as Hodgkin or non-Hodgkin lymphomas. The malignant proliferation of lymphoid cells mainly occurs in lymph nodes, but in a small number of cases, it can be extranodal. The oral cavity represents a very rare primary extra-nodal location for non-Hodgkin lymphoma and can pose a diagnostic challenge for the dentist.

We report the case of a woman in her 50s presenting a primary oral manifestation of a diffuse large B-cell lymphoma (DLBCL). The patient was complaining of an inflammatory swelling of the vestibular gingiva of the left maxillary premolars, which had been growing for four months. She had consulted several dentists who suspected a dental cause, without any clear argument in favor of a periodontal or endodontic etiology. Periodontal treatments were initiated, and multiple courses of antibiotics were prescribed without improvement in the clinical presentation. Finally, the patient was referred to the hospital where a biopsy of the gingival lesion was performed. Histological and immunohistochemical examinations led to the diagnosis of a DLBCL. The patient was referred to a hematology-oncology department and received polychemotherapy treatment until complete remission.

Initial oral manifestations of lymphoma may mimic dental, endodontic, or periodontal pathology. Appropriate oral clinical and radiological examinations should exclude local causes. If these examinations are inconsistent with the clinical presentation, systemic diseases should be considered, and a prompt biopsy of the lesion should be performed to obtain a definitive diagnosis. Early detection of a primary oral lymphoma can improve the prognosis with timely multidisciplinary medical management.

## Introduction

Non-Hodgkin lymphomas are malignant proliferations of B or T lymphocytes and form a highly heterogeneous group of tumors classified into various histological types on the basis of histopathologic, cytogenetic, and molecular biological analysis [1,2]. B lymphocytes are involved in 85% of cases and T lymphocytes in only 15% of cases [1,2]. Diffuse large B-cell lymphoma (DLBCL) is one of the most common aggressive forms, regardless of the location [2,3].

Cervical lymphadenopathy is the most frequent clinical manifestation, but 20 to 30% of cases may be extranodal [1,2]. The most commonly affected extranodal sites are the spleen, liver, gastrointestinal tract, skin, lung, central nervous system, and orbits [1,2]. Non-Hodgkin lymphoma comprises approximately 5% of head and neck malignancies, and occurs, with or without lymph node involvement, in areas such as Waldeyer's ring (i.e., the tonsils, pharynx, and base of the tongue), salivary glands, orbit, nasal cavity, paranasal sinuses, and thyroid gland [2,4,5]. Extranodal non-Hodgkin lymphomas of the oral cavity and the maxillofacial region can affect soft and/or hard tissues and present as nodules or swellings with or without surface ulceration, intermittent pain, unexplained dental mobility, nasal congestion, pharyngeal foreign body sensation, dysphagia, or odynophagia [5-15].

Primary non-Hodgkin lymphomas of the oral cavity with exclusive soft tissue involvement, without underlying bone invasion, are very rare [8,11-14], and can represent a diagnostic challenge for the untrained dentist, who must be able to consider a general etiology, after ruling out a dental cause.

In this article, we report the case of a patient presenting a DLBCL with a single gingival localization, mimicking a dental abscess, leading to diagnostic and therapeutic delays of several months.

## Case presentation

A 53-year-old female patient was referred in July 2023 to the oral surgery department of a French public university hospital by her primary care dentist for specialized advice. She did not have any current or past history of alcohol or tobacco use. Her medical history included dyslipidemia treated with rosuvastatin, and moderate thrombocytopenia (platelet count between 100 and 150 G/L) since 1985, which had never been investigated. The patient was postmenopausal and took hormone replacement therapy (progesterone).

She presented a gingival swelling that had been gradually increasing for approximately four months, located on the buccal gingiva of the left maxillary premolars and first molar. The patient reported constant functional discomfort during chewing and speaking, as well as occasional moderate pain that responded to acetaminophen. No sign of deterioration of the general status was reported. She had consulted several dentists, including a general dentist, an endodontist, and a periodontist, who successively considered an endodontic or periodontal abscess despite the lack of convincing clinical or radiological evidence. Multiple courses of antibiotics were prescribed, without significant impact on the lesion course.

**Figure 1:**
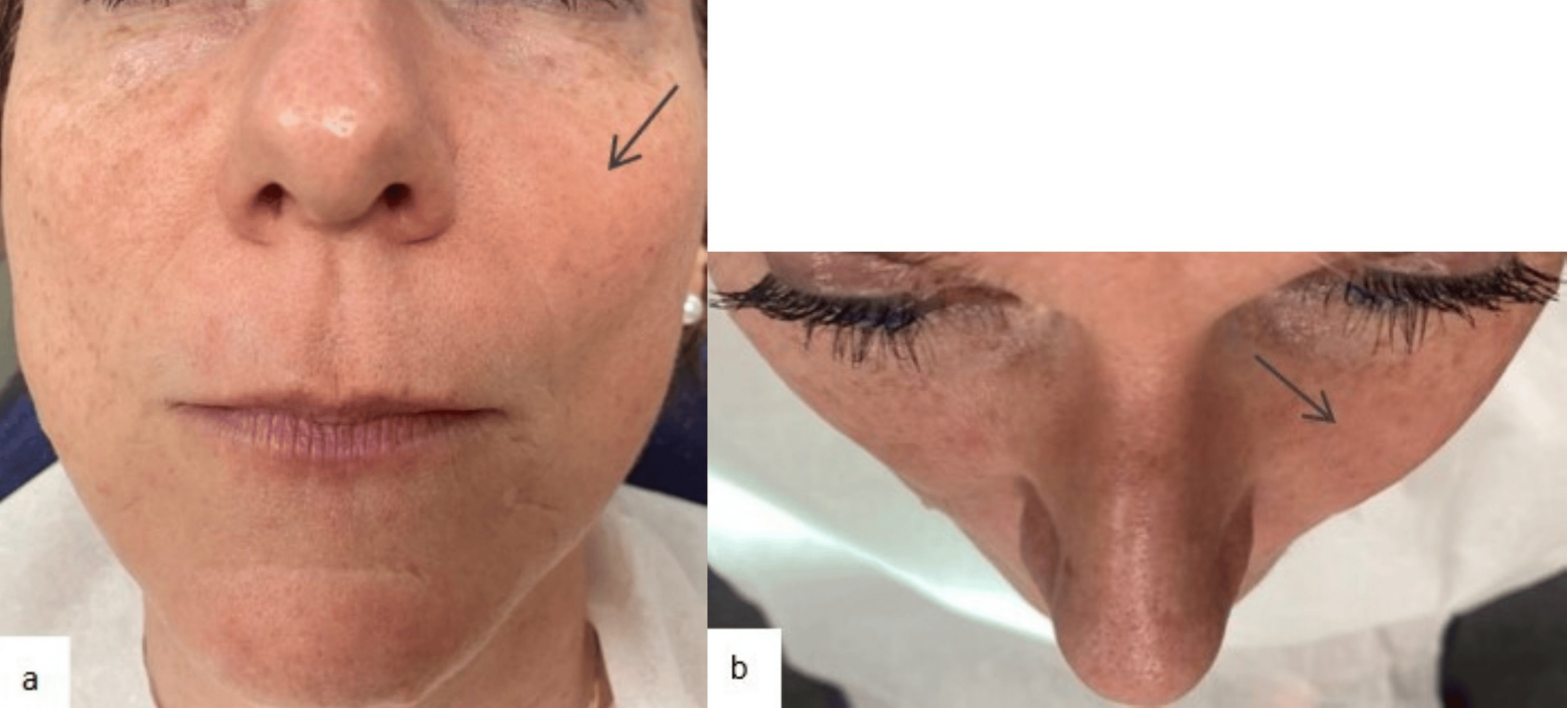
Extraoral views. a: Front view of the patient showing facial asymmetry due to left cheek and para-nasal swelling. b: Top view of the patient showing facial asymmetry. The black arrows show the facial asymmetry.

An extraoral examination revealed a left cheek and para-nasal swelling that was continuous with the anterior wall of the maxillary bone, covered with normal-appearing and mobile skin. No sensory-motor dysfunction or cervicofacial lymphadenopathy was noted. The patient reported a sensation of local pressure that had worsened over time (Figure 1).

**Figure 2:**
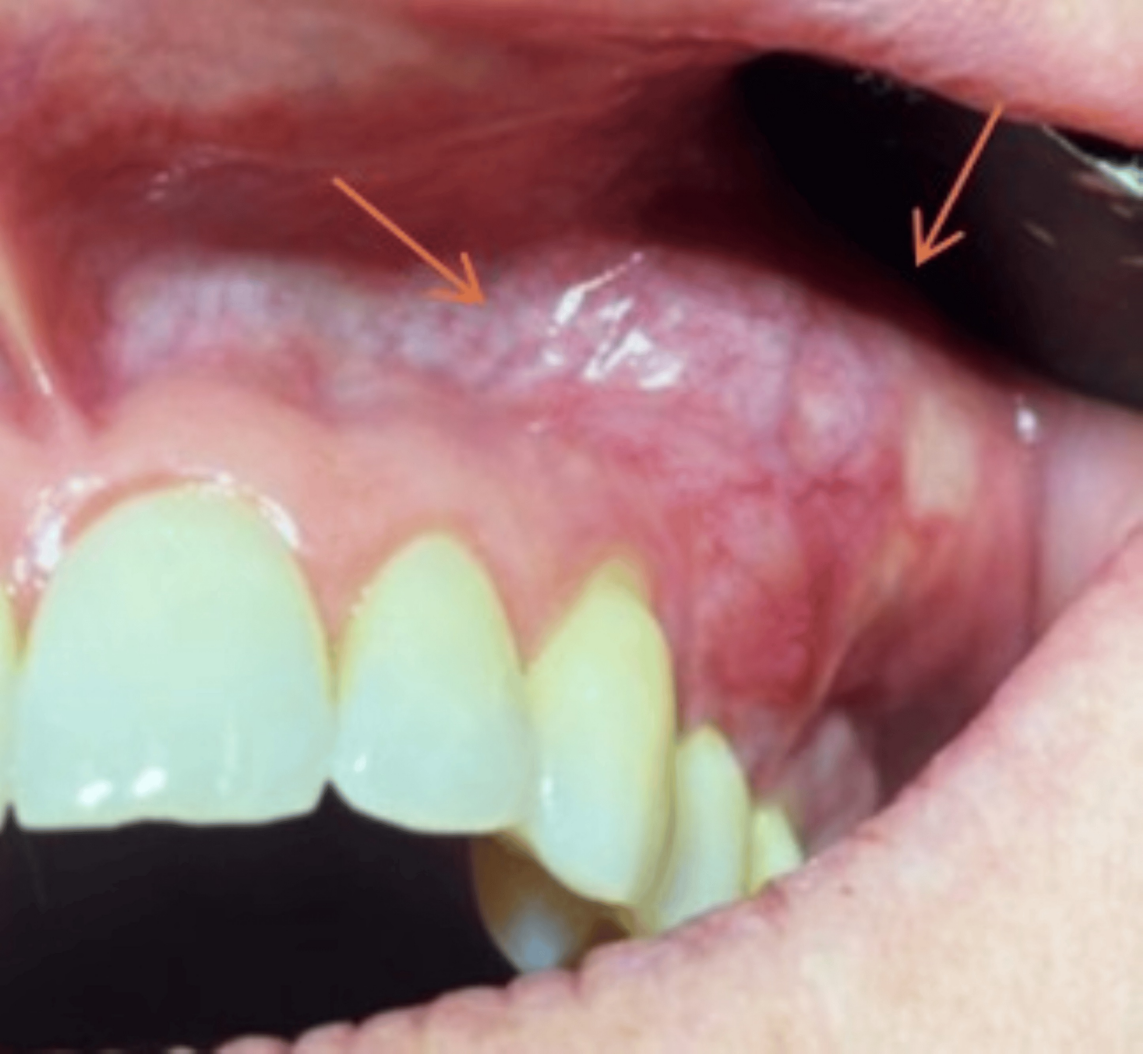
Intraoral view. Left maxillary lesion at the level of the gingiva and in the depth of the buccal vestibule, extending from the canine to the first molar. Orange arrows show the tumor’s limits.

An intraoral examination revealed good oral hygiene. The teeth in the left maxillary region had physiological mobility, and both axial and transverse percussion were painless. Periodontal probing was less than 3 mm, and vestibular palpation caused minimal discomfort. A pulp sensitivity testing yielded positive results for all teeth, except for the first and second molars, which were endodontically treated and crowned. In terms of mucosal appearance, there was a well-defined swelling with an irregular, bumpy surface, measuring approximately 2 cm in mesiodistal length, located in the area of the canine, premolars, and first molar. The swelling was situated on the gingiva and in the depth of the buccal vestibule. The overlying mucosa displayed an erythematous appearance with a well-defined vascular pattern (Figure 2).

**Figure 3:**
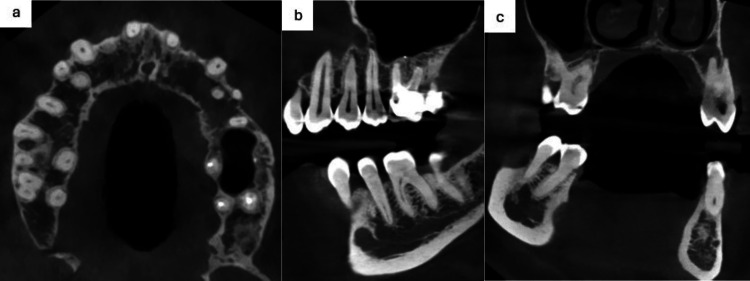
Cone beam computed tomography (CBCT). a: Axial section showing the relationship between the roots of the left-sided teeth and the maxillary alveolar bone, and the flaky aspect of the maxillary bone. b: Sagittal section that raised the suspicion of a radiolucent lesion on the first premolar’s apex showing the absence of the left maxillary sinus involvement and the atypical aspect of the spongious bone of the jaws. c: Coronal section showing the persistence of palatal and vestibular cortical bone and the blunted aspect of the apices of the first premolar.

The patient brought the cone beam computed tomography (CBCT) performed by the general dentist (Figure 3). He suspected radiolucent images at the apices of the first premolar, which appeared blunt, and therefore erroneously concluded a dental cause.

In our opinion, the CBCT did not reveal any bone-related changes underlying the mucosal lesion, apart from a low bone density of the spongious bone with sparse trabeculations throughout the jawbones. The cortical bone layers were intact. There was no involvement of the maxillary sinus.

Because the clinical presentation and dental and radiographic examinations did not support an endodontic or periodontal etiology or a primitive bone pathology, a soft tissue tumor in the peri-maxillary area was suspected. A blood panel, magnetic resonance imaging (MRI), and a computed tomography (CT) scan of the facial area with and without contrast injection were prescribed.

The blood panel revealed a platelet count of 106 G/L and a decreased glomerular filtration rate of 67 mL/min/1.73m², indicating mild renal insufficiency. Serological tests for hepatitis B, hepatitis C, and HIV were all negative, and inflammatory biomarkers were within normal ranges.

**Figure 4:**
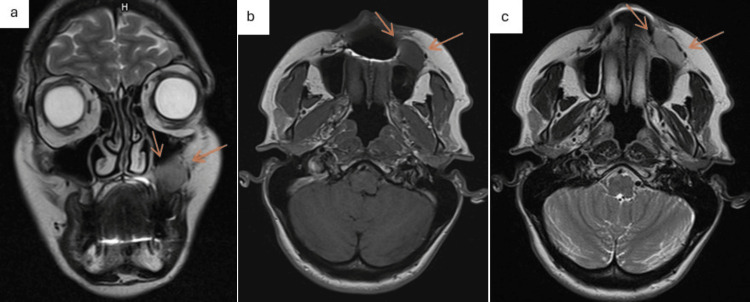
Facial magnetic resonance imaging (MRI). a: Non-injected, T2-weighted sequence, coronal section showing the hypersignal lesion. The lesion pushes back the sinus floor without invading it. b: Non-injected, T1-weighted sequence, axial section, showing the hyposignal lesion ahead of the anterior wall of the left maxillary sinus. c: Injected, T2-weighted sequence, axial section, showing the lesion ahead of the anterior wall of the left maxillary sinus; not readily interpretable contrast of lesion due to artifacts caused by dental material. Orange arrows indicate the tumor's extent.

MRI findings indicated a lesion located in front of the anterior wall of the left maxillary sinus, in its lower part. The lesion was oval with regular contours, measuring 25 mm * 15 mm (axial section) * 20 mm (height in coronal section) and extending to the left buccinator muscle. No lymphadenopathy was noted (Figure 4). The contrast uptake of the lesion could not be interpreted due to artifacts from dental materials.

The facial CT scan confirmed the presence of the lesion with regular contours located in front of the left maxilla and within the superficial fascia. There was no invasion of the adipose tissue, the masticatory space, or the masseter muscle.

**Figure 5:**
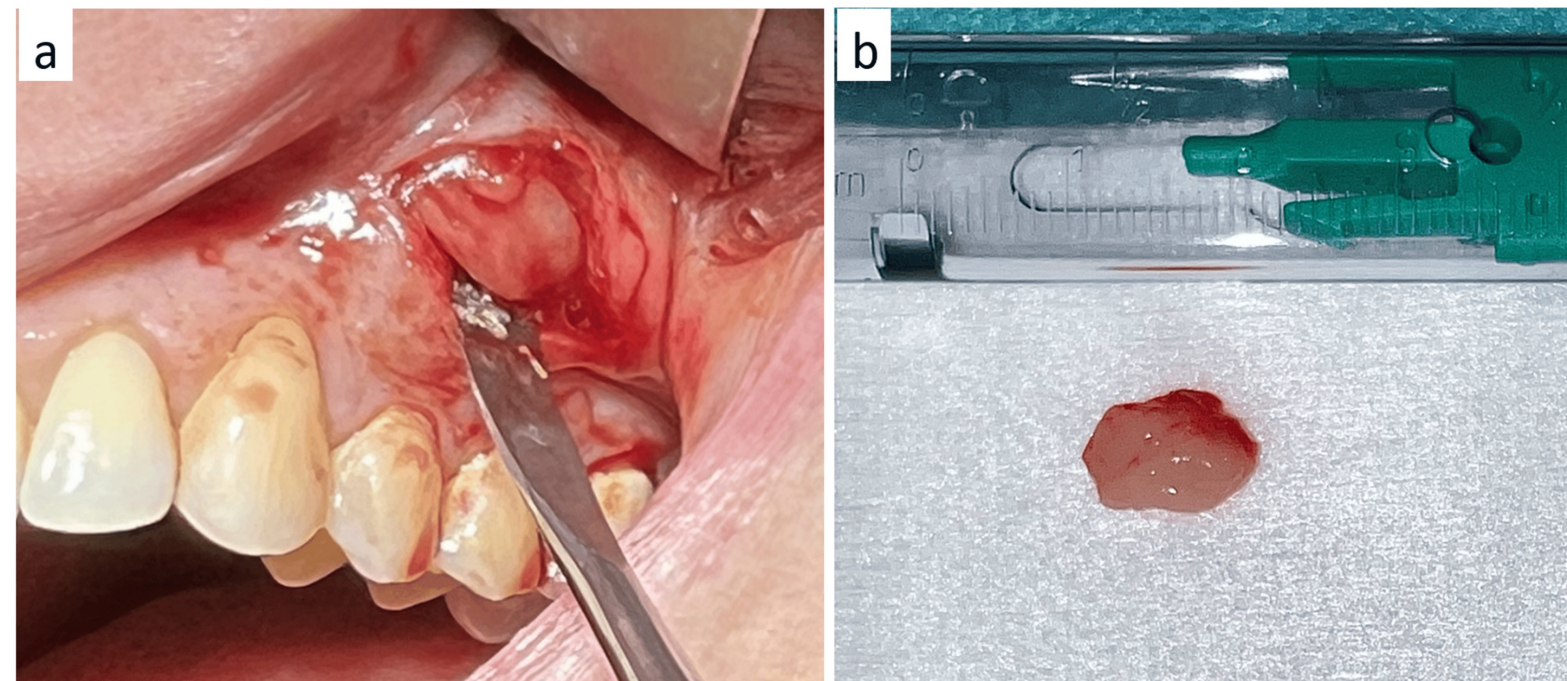
Intra-operative views of the biopsy. a: Well-encapsulated lesion, presence of cleavage planes. b: Macroscopic aspect of the biopsy specimen (whitish and avascular tissue).

Written informed consent was obtained at the first appointment in the oral surgery department, and the biopsy was realized at the second appointment after obtaining the patient's consent. The biopsy of the lesion was performed under local anesthesia and revealed a well-encapsulated lesion, with a cleavage plane, whitish appearance, and firm consistency (Figure 5).

**Figure 6:**
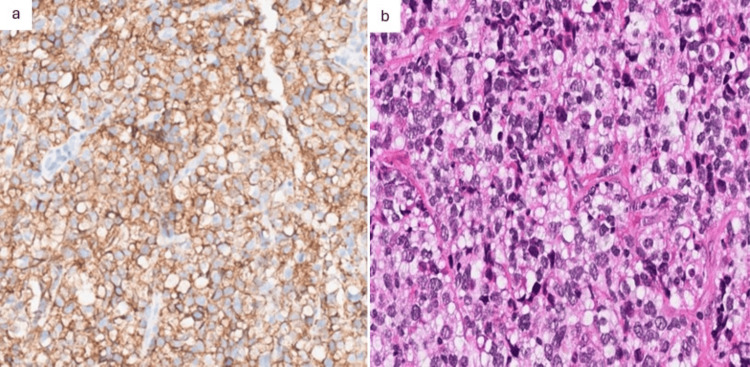
Histological sections on paraffin-embedded samples. a: Immunohistochemical staining at magnification 20x positive for CD20. b: Hematoxylin-eosin-saffron (HES) staining at magnification 20x: diffuse large B-cell proliferation.

The histopathological analysis, along with immunohistochemical staining, revealed a diffuse large B-cell lymphoma of germinal center type. This was characterized by a proliferative pattern of organized lymphoid tumor cells in diffuse sheets of large cells with irregular nuclei, numerous mitoses, and staining CD20+, CD5-, CD10+, BCL6+/-, MUM1+, and BCL2+ on the tumor cells (Figure 6). The Ki67 proliferation index was approximately 50%.

The staging evaluation of the malignant hemopathy, including a positron emission tomography (PET) scan and a cervico-thoracic-abdominal-pelvic CT scan, revealed no other lymph node or extranodal involvement and concluded that the DLBCL was localized exclusively in the oral cavity.

Following the diagnosis, the patient was urgently referred to a specialized oncology and hematology center. She received chemotherapy according to the R-CHOP protocol (rituximab plus cyclophosphamide, doxorubicin, vincristine, and prednisone) administered in four cycles spaced three weeks apart and achieved a complete remission.

## Discussion

Non-Hodgkin lymphoma is an extramedullary malignant lymphoid proliferation that initially affects primarily lymph nodes and the entire lymphoid system but any organ may be concerned. From a histopathological perspective, the diagnosis is made in the absence of Reed-Sternberg cells (pathognomonic for Hodgkin lymphoma) and the presence of other neoplastic B or T lymphoid cells, which can be more or less differentiated [1,2,4]. It is one of the most common diagnoses in cases of chronic, painless cervical or axillary lymphadenopathy, blood count abnormalities (e.g., anemia, thrombocytopenia, peripheral lymphocytosis, hypogammaglobulinemia), or systemic symptoms (fever, night sweats, weight loss) [1,2,4].

In Western countries, the incidence is 14 to 19 cases per 100,000 person-years, and it has been increasing at a rate of 3-4% per year since 1970 [2]. In France, there has been a significant increase in the number of incident cases (82% increase between 1995 and 2018, with a total of 5,071 new cases in 2018) [16]. The median age at diagnosis is 69 years for men and 71 years for women, with a male-to-female ratio of 3:2 [2,16]. The patient's medical context greatly influences the development of this disease [1,2,4]. Immunosuppression, bacterial infections (Helicobacter pylori or Chlamydia trachomatis) or viral infections (HIV, hepatitis B virus (HBV), hepatitis C virus (HCV), human herpesvirus-8 (HHV-8), Epstein-Barr virus (EBV), human T-lymphotropic virus type 1 (HTLV-1), and human papillomavirus (HPV)), and exposure to ultraviolet radiation, pesticides, wood dust, organic solvents, and dioxin seem to increase the risk of developing the disease [1,2,4]. However, in most cases, the etiology is not found, as in the reported case. The most common non-Hodgkin lymphoma subtypes by far in developed countries are DLBCL (about 30%) and follicular lymphoma (about 20%) [17].

Oral involvement is extremely rare (2-3% of extra-nodal cases), and the most commonly involved sites are the maxilla (especially soft palate) (77%), accessory salivary glands, tonsils, and less frequently gums and periapical areas [2,4,5].

A few cases similar to ours have been reported in the literature. Kuceki et al. [11] and Kobler et al. [13] reported two cases of DLBCL with unique gingival localization, without underlying bone lesion nor adenopathy or other extra-nodal involvement. In the case report of Kuceki et al. [11], the lesion appeared as a maxillary gum swelling on the vestibular and palatal side, evolving for three months and mimicking a dental abscess. Therefore, the dentist prescribed doxycycline without a controlled examination. In the face of antibiotic inefficiency, the patient decided to consult a dermatologist, who performed a punch biopsy. A histopathologic analysis revealed an atypical lymphocytic infiltrate with a mixture of B and T cells, without a precise diagnosis. The patient was then referred to an otorhinolaryngologist for a larger and deeper incisional biopsy, wherein immunostaining concluded a DLBCL.

In the case report of Kobler et al. [13], the lesion appeared as an inflammation of the vestibular gingiva near an infected and painful residual root, mimicking a dental abscess. The radiographic examination was unremarkable. The general dentist referred the patient to an oral surgeon, who performed the biopsy at the same time as the root extraction, which led to the diagnosis.

Kolokotronis et al. [14] reported four cases of primary oral DLBCL, three localized within the palate and one within the cheek, all without evidence of bone invasion or adenopathy.

Vinoth et al. [15] reported the case of a 14-year-old girl presenting multiple gingival swellings evolving for two months. She was initially treated for suspected gingivitis, but the swelling still progressed. She had no medical history or adenopathy. The dentist performed a biopsy, which revealed a lymphoproliferative disorder. She was then referred to the hospital for a second biopsy, which was consistent with the diagnosis of DLBCL. Extension work-up revealed an involvement of the left masticatory space with an erosion of the postero-lateral wall of the left maxillary sinus and the extension within it.

Parihar et al. [12] reported the case of a woman in her 50s who presented an anterior mandibular gingival soft, sessile, and friable mass, bled on palpation, displacing teeth and involving mandibular labial sulcus, with subjacent bone lesion and paresthesia in the chin area. It had been evolving for more than 14 months. She had no systemic symptoms or adenopathy. Her dentist initially thought of a pyogenic granuloma and excised the lesion in conjunction with antibiotic therapy. The lesion recurred six months later, involving the whole area between the two mandibular canines. A new excisional biopsy was performed, which led to the diagnosis of DLBCL.

According to the Cotswolds-modified Ann Arbor classification [1,2,4], our patient was at stage IE (extra-nodal localization limited to a single site), sub-stage A due to the absence of general symptoms (i.e., weight loss, fever, or night sweats). Similarly to the reported case, none of the cases with the primary oral location of the DLBCL described above presented signs of alteration of the general status.

Non-Hodgkin lymphomas can be indolent (slow-growing, poorly responding to treatment, generally not curable), or very aggressive (fast-growing, responsive to chemotherapy, and often curable) [1,2,4]. B-cell lymphomas have a better prognosis than T-cell lymphomas, and when managed early, have a good five-year survival rate (70 to 100% for single-location cases) [2-5,18]. In a retrospective study, Mian et al. [18] reported 488 cases of extra-nodal DLBCL (24 in the oral cavity), with a complete response to treatments in 91% of cases, recurrence at five years in only 9% of cases and five-year survival rate around 90%.

Poor prognostic factors for lymphomas include age over 60 years, stage III or IV, involvement of the bone marrow, involvement of more than one extra-nodal site, high proliferation index Ki67 over 90% (immunochemistry), high lactate dehydrogenase level, poor patient performance status, and anemia [1-4,19]. Consequently, the prognosis worsens with the number of these factors. Patients with four or five risk factors have a complete response rate to treatment of approximately 44% and a five-year survival rate of approximately 26% [2]. Patients with no risk factors have a rate of complete response of approximately 87% and a five-year survival rate of approximately 73%, with a very high cure rate. For the DLBCL (considered an aggressive form), a complete response to treatment is expected in 80% of cases, with a complete cure rate of around 60% [2-4]. In the reported case, the patient presented a DLBCL, without any poor prognostic risk factors and a Ki67 index of 50%, which predicted a favorable outcome.

Diffuse large B-cell lymphomas are conventionally treated with R-CHOP chemotherapy, which combines rituximab (anti-CD20), cyclophosphamide, doxorubicin, vincristine, and prednisone [1,2,4,19]. Adjuvant radiotherapy (40-50 Gy) may be added [2,5,19] and, in some very aggressive cases, autologous stem cell transplantation may be considered [2].

In the case reported by Kobler et al. [13], the patient achieved clinical remission in 11 months after eight cycles of CHOP and adjuvant radiotherapy. In the case reported by Kuceki et al. [11], the PET scan showed complete remission after three cycles of R-CHOP and the patient underwent consolidative radiotherapy. In the case reported by Parihar et al. [12], the complete response was achieved with four cycles of R-CHOP and radiotherapy. In the four cases of oral DLBCL reported by Kolokotronis et al. [14], one was treated with surgery and chemotherapy (palate, stage IE). He relapsed at 18 months and died. Another one was treated with chemotherapy (cheek, stage IE) and was free of disease at 38 months of follow-up. Another woman was treated with chemotherapy (palate, stage IIIE (nodal groups on both sides of the diaphragm with localized extranodal site)); she relapsed but then was free of disease at 30 months of follow-up. Finally, the last woman received chemotherapy (palate, stage IE) and was lost to view at three months. In our patient, the response to chemotherapy was complete after four courses, and no additional treatment was administered. No relapse was noted after 14 months of follow-up. 

In the reported case, there was a four-month delay before the definitive diagnosis. The discrepancy between symptoms, objective clinical signs, dental and periodontal diagnostic tests, and radiological findings should have prompted healthcare professionals to perform a biopsy earlier, which would have allowed for quicker oncologic and hematologic management.

## Conclusions

The clinical and radiological presentation of DLBCL is highly variable. Dentists must be aware of this disease to avoid significant diagnostic delays. When faced with orofacial abnormalities that mimic dental pathologies but the causative tooth is not found after a thorough clinical examination and conventional diagnostic tests, clinicians should consider a systemic disease with primary oral manifestations. An early histopathologic examination allows for a prompt definitive diagnosis.
